# Effect of Light Treatment on Chemical Composition of *Andrographis paniculata* Seedlings

**DOI:** 10.3389/fchem.2022.889365

**Published:** 2022-07-05

**Authors:** Zhehui Jiang, Tianhao Wang, Kaixue Zhang, Meiqi Zhang, Ling Ma, Weichao Ren, Yihong Bao, Wei Ma

**Affiliations:** ^1^ College of Forestry, Northeast Forestry University, Harbin, China; ^2^ College of Mechanical and Electrical Engineering, Northeast Forestry University, Harbin, China; ^3^ State Grid Tangshan Power Supply Company, Hebei, China; ^4^ College of Pharmacy, Heilongjiang University of Chinese Medicine, Harbin, China; ^5^ Key Laboratory of Forest Food Resources Utilization of Heilongjiang Province, College of Forestry, Northeast Forestry University, Harbin, China

**Keywords:** light quality, *Andrographis paniculata*, chemical composition, secondary metabolism, active ingredient

## Abstract

Light quality consists of a spectrum of different bands, which not only affects plant, development, and primary metabolism but also affects the secondary metabolism of plants. It is an important factor affecting the content of active components of medicinal plants. The *A. paniculata* seedlings planted in the laboratory, as materials, were tested with red light, far red light, blue light, and ultraviolet light separately. The study assays the content of six main chemical components separately by LC-MS, observes the changes in the content, and analyzes the relationship between the light quality and the active ingredient of *A. paniculata*. Using the ointment yield and pH value, the fingerprint analysis method of *A. paniculata* standard decoction was established, and we discussed the selection of index components of *A. paniculata* standard decoction. It was suggested to select andrographolide as the index component. It will provide a theoretical basis for the large area cultivation of *A. paniculata* and optimize the quality of medicinal materials to ensure the quality of standard decoction.

## 1 Introduction

Light is one of the most important factors in plant growth. Plants convert light energy into chemical energy through photosynthesis and store light energy in the body to provide energy for their own growth (Yi Wen and Shi, 2018; Gupta and Nath, 2020). But different plants have different sensitivities to light, as do medicinal plants (Zou et al., 2020; Lv et al., 2021). According to the different needs of medicinal plants for light, it can be divided into three categories: sunny plants such as *Rehmannia* and licorice; shade plants such as *Panax notoginseng* and ginseng; and intermediate plants with moderate light need (Wu, 2013), such as *Ophiopogon japonicus* and *Viola philippica*. Related studies have shown that light quality affects the growth and development of plants; on the other hand, it has a greater impact on the primary and secondary metabolites of plants (Li, 2006; Zhang, 2010; Chen, 2012; Gao, 2012). At the same time, different wavelengths of light have different effects on the plant secondary metabolism, and the light of the same wavelength also has different responses to different plants. Blue light can promote the accumulation of matrine and oxymatrine (Wang, 2013); the content of total triterpenoids in *Ganoderma lucidum* spore powder is not obvious, but blue light contributes to the accumulation of polysaccharides in *Ganoderma lucidum* (Hao, 2011); both ultraviolet and blue light affect the synthesis of anthocyanin in lettuce leaves, while blue light also contributes to the synthesis of carotenoids in lettuce leaves. The total phenolic acid content in red-light-treated lettuce leaves is high (Li, 2010); blue light can effectively increase the content of total alkaloids in the tuber of *Pinellia ternata*, but green light is the opposite (Hu, 2013); blue light and green light can promote the synthesis of total flavonoids, polysaccharides, and ferulic acid of *Anoectochilus roxburghii*, and red light can increase the production of polysaccharide (Liu, 2013). Most of the current literature on light quality is research on agricultural production, and it has achieved certain results (Rehman et al., 2020; Li et al., 2021). There are few research studies as for the cultivation of Chinese medicine (Qi, 2007; Guo et al., 2011; Zhang et al., 2014; Liu and Liu, 2016).

*Andrographis paniculata* is a common Chinese medicine for clearing away heat and detoxification, and its diterpene lactone component is the main active ingredient. There are more than 10 kinds of Chinese patent medicine preparations, including capsules, dripping pills, and tablets, which use *A. paniculata* as the monarch drug. *A. paniculata* as medicinal material is widely used in clinical practice with high annual demand. There are many research works on its cultivation techniques, but there are few research reports on the growth, chemical composition, and quality of *A. paniculata* involving light quality.

The high-throughput liquid chromatography with mass spectrometry (LC-MS) method could be used for the rapid characterization of multiple chemical constituents and metabolites of herbal medicine (Sun et al., 2012; Sun et al., 2013; Yan et al., 2013; Zhang et al., 2018; Zhang et al., 2019). This study was designed to determine the effect of light treatment on the chemical composition of *A. paniculata* seedlings, used the laboratory cultured *A. paniculata* seedlings as research objects, and detected six components using LC-MS. It carried out extensive research into the effects of ultraviolet light and other light treatments on the six components of *A. paniculata* seedlings, and provides reference for the research on the large-scale planting of *A. paniculata* and the light quality of other Chinese medicinal materials.

In addition, 12 batches of *A. paniculata* decoction pieces from different origins were used as samples, and the standard decoction of *A. paniculata* was prepared according to the process of the whole herbal medicinal materials in the “Research Strategy for Standard Decoction of Chinese Herbal Decoction Pieces” proposed by Chen et al. (2016). Using the ointment yield and pH value, the fingerprint analysis method of *A. paniculata* standard decoction was established, and the selection of index components of *A. paniculata* standard decoction was discussed. It was suggested to select andrographolide as the index component. The establishment of the standard provides a reference.

## 2 Materials and Methods

### 2.1 Materials

Andrographolide reference substance (Ap, purity: 98.0%, batch number: BBP60056), neoandrographolide reference substance (Neo, purity: 98.0%, batch number: BBP02282), 14-deoxyandrographolide reference substance (30, purity: 98.0%, batch number: BBP02369), 14-deoxy-11-hydroxyandrographolide reference substance (S4, purity: 95.0%, batch number: BBP02272), and 14-deoxy-17-hydroxyandrographolide reference substance (S7, purity: 95.0%, batch number: BBP02304) were purchased from Yunnan Xili Biotechnology Co., Ltd. Reference substance 14-deoxyandrographolide (S3, purity: 98.0%, CAS number: 79233-15-1) was purchased from Shanghai Yuanye Biotechnology Co., Ltd. Methanol and cetonitrile were chromatographically pure (Fisher, United States). Formic acid (chromatographic grade, purity 98.0%) and ammonium formate (chromatographic grade, purity 99.0%) were provided by Shanghai Aladdin Biochemical Technology Co., Ltd. Water used was purified water.

A total of 12 batches of *A. paniculata* were from five regions in Guangxi, Fujian, Anhui, Hubei, and Guangdong Province. The research samples were identified by Sun Wei, associate researcher of the Institute of Chinese Materia Medica, Chinese Academy of Chinese Medical Sciences, and the research samples were *A. paniculata* (Burm. f.) Nees. The detailed origin information is shown in Table 1 in the following text.

**TABLE 1:** Ion information table of reference substance.

Reference substance	Parent ion	Child ion 1	Child ion 2	Collision energy (child ion 1)	Collision energy (child ion 2)	Voltage (V)
				(V)	(V)	
Neo	498.4	319.6	301.6	10	18	130
30	497.3	317.3	259.3	6	12	100
S3	335.2	299.3	287.3	8	10	100
S4	373.2	149.1		19		135
S7	353.2	299.3	287.3	15	15	130

Take the appropriate amount of *A. paniculata* seeds, sow them in a rectangular flowerpot, and then cover them with plastic film for 6 days to facilitate the emergence. Place the planted flowerpots in the plant culture room at a culture temperature of 25°C, with about air humidity of 15% and light for 24 h every day; and water regularly until the seedlings grow to the first pair of true leaves (the results of the pre-experiment showed that the chemical contents of the leaves were more balanced in this period), and then do the further research.

### 2.2 Methods

#### 2.2.1 Chromatography-MS Conditions

The experiment used Agilent ultra-performance liquid chromatography–tandem mass spectrometry (UPLC-QQQ-MS), an external standard method to calculate the concentration, using multiple reaction monitoring mode (MRM) detection (American Agilent UPLC-MS system, including 1,290 series ultrahigh performance liquid chromatography and 6,470 triple quadrupole mass spectrometer). Column: Agilent ZORBAX XDB-Eclipse Plus C18 (2.1 mm × 50 mm, 1.8 μm); ion source was electrospray ion source (AJS ESI source), the mobile phase was 0.1% formic acid 5 mmol aqueous ammonium formate (A) acetonitrile (B); Ap gradient elution conditions in negative ion mode: 0–3 min, 5%–70% B; 3–4 min, 70%–100% B; 4–6 min, 100% B; temperature 35°C; injection volume 1 μl; flow rate 0.3 ml/min. The precursor ions obtained by scanning were 395.2, the daughter ions were 331.4 and 287.0, the collision energies were 7 and 15 V, respectively, and the voltage was 100 V. In the positive ion mode, the other five reference gradient elution conditions are as follows: 0–2 min, 6%–25% B; 2–6 min, 25%–40% B; 6–7 min, 40%–80% B; 7–10 min, 80%–100% B; 10–11 min, 100% B; column temperature 30°C; the sample volume was 1 μl; and the flow rate was 0.3 ml/min.

#### 2.2.2 Ultraviolet Light and Other Light Processing Methods

The same batch of seedlings was taken for the treatment of *A. paniculata*, which were divided into ultraviolet light group and dark group, placed in ultraviolet light (light intensity was 1) and dark place, and treated for 5 h at the same time. Each group needs three repetitions, and eight seedlings were required for each repetition.

The same batch of seedlings was taken for the treatment of *A. paniculata* and divided into the red light group, blue light group, far-infrared group, and dark group; placed in red light (light intensity was 62), blue light (light intensity was 59), far-infrared light (light intensity was 2), and darkness; and processed for 48 h. Each group needs three repetitions, and eight seedlings were required for each repetition.

After the treatment, the flowerpots were equally divided into eight regions, one seedling was selected for each region, and the leaves of the seedlings were all cut with scissors, including a small part of stems. The sampling speed should be fast so as to avoid the sample being placed for a long time and affecting the chemical content. When selecting the seedling, try to choose the seedling with the same growth. The numbered samples should be immediately frozen in liquid nitrogen and stored in a −80°C refrigerator.

#### 2.2.3 Preparation of Test Solution

The sample taken out from the refrigerator at −80°C was immediately placed in liquid nitrogen and ground into powder to prevent samples from absorbing moisture. Before the sample absorbs moisture, 0.1 g of the powder was quickly weighed using the electronic analytical balance (one in 10,000, Beijing Sartorius Balance Co., Ltd.) and placed directly in a 50-ml centrifuge tube; 10 ml of methanol was weighed by measuring cylinder and added in the tube of the sample, vortexed for 30 s, and ultrasonicated for 30 min. Then the centrifuge tube was placed in a 4°C refrigerator for 8 h or overnight, centrifuged by the Scanspeed mini high-speed centrifuge (Labogene, Denmark) at 1880 rpm for 10 min, and the supernatant was taken to get the sample solution.

Accurately 1 ml of each of the above solutions was drawn into a 2-ml centrifuge tube, and filtered with a 0.22-um filter. The prepared test solution was diluted 10 times with methanol before the measurement of the content of Ap.

#### 2.2.4 Preparation of Reference Stock Solution

The appropriate amount of Ap, Neo, 30, S3, S4, and S7 reference substances was accurately weighed, and they were placed in a brown vial to prepare reference stock solution separately. Each reference substance stock solution concentrations were 1.0 mg/ml, 0.43 mg/ml, 0.50 mg/ml, 0.83 mg/ml, 0.35 mg/ml, and 0.20 mg/ml.

#### 2.2.5 Standard Curve Preparation

Ap reference substance stock solution was taken, diluted with methanol, and a series of controls with concentrations of 104 ng/ml, 2,000 ng/ml, 1,000 ng/ml, 500 ng/ml, 200 ng/ml, and 100 ng/ml was prepared. In accordance with the chromatographic conditions of the negative ion mode in Section 2.2.1, 1 µl of the solution was extracted from small to large and injected into the liquid chromatography mass spectrometer. The concentration was used as the abscissa and the peak area was used as the ordinate to obtain the standard curve. The regression equation was Y = 4.8912X-286.24 (R^2^ = 0.999), and the linear range was 10^2^∼10^4^ ng/ml.

In total, 100 µl each of Neo, 30, S3, S4, and S7 reference substance stock solutions were taken; they were placed in the same vial and mixed thoroughly. This mixed reference solution was diluted by 2, 5, 10, 20, 50, 100, 200, 500, 1,000, 2000, 5,000, 10,000, and 20,000 times. According to the chromatographic conditions of the positive ion mode in Section 2.2.1, 1 µl of the reference substance solution was accurately absorbed and injected into the liquid chromatography–mass spectrometer. Based on the peak area results of the sample and the reference substance, six concentrations were selected so that the concentrations of all samples were included in the six concentration ranges. The concentration was taken as the abscissa and the peak area as the ordinate to obtain the standard curve.

#### 2.2.6 Sample Content Determination

In total, 1 µl of the test solution was separately absorbed and diluted 10 times, and injected according to the chromatographic conditions of the negative ion mode in Section 2.2.1 to obtain the amount of Ap. Then 1 µl of the test solution was accurately drawn and injected according to the chromatographic conditions of Section 2.2.1 in the positive ion mode to obtain the amount of the other five contents (Neo, 30, S3, S4, and S7).

### 2.3 Preliminary Study on Quality Standard of *A. paniculata* Standard Decoction

#### 2.3.1 Origin Identification

The sample base identification was carried out according to the standard procedure of DNA barcode molecular identification (Chen et al., 2013). Each batch of *A. paniculata* samples was taken, crushed with a ball mill, and about 30–40 mg was taken, washed with nuclear separation liquid 3–5 times until the supernatant was colorless, and then used a plant genome extraction kit to extract DNA, and the obtained DNA was subjected to ITS2 sequencing PCR amplification: ITS2 forward and reverse primers were 2F (5'-ATG​CGA​TAC​TTG​GTG​TGA​AT-3') and 3R (5'-GAC​GCT​TCT​CCA​GAC​TAC​AAT-3'); the total PCR reaction system was 25 μl, including 2 × Taq PCR Master Mix (Beijing Adelaide Biotechnology Co., Ltd.) 12.5 μl, forward and reverse primers 1 μl (2.5 μM) each, DNA template 2 μl, and the rest were double-distilled water; the amplification program was: 94°C pre-denaturation for 5 min, 94°C denaturation for 30 s, 56°C annealing for 30 s, 72°C extensions for 45 s, a total of 40 cycles, and the last 72°C extension for 10 min. The PCR amplification results were detected by agarose gel electrophoresis, and the samples showing the bands were sequenced in two directions. The sequencing results were spliced using CodonCode Aligner V5.1.5 software to obtain the complete ITS2 sequence. Finally, the complete sequence was submitted to the following identification website (http://www.tcmbarcode.cn/china/) for comparison and identification. The 12 batches of *A. paniculata* samples were identified by DNA barcoding technology, and they were all from *A. paniculata* (Burm. f.) Nees, which was in line with the 2015 edition of the Chinese Pharmacopoeia on the original plant of *A. paniculata*.

#### 2.3.2 Preparation of Standard Decoction

Chen et al. (2016) in total, 100 g of *A. paniculata* pieces was taken from each batch; they were put in a 2000-ml round-bottomed flask, added 12 times the amount of water to soak for 30 min, heated under reflux for 30 min, and filtered while hot, and then 10 times the amount of water was added to the dregs, heated under reflux for 20 min, and filtered while hot. The two filtrates were combined and concentrated under reduced pressure to 500 ml.

#### 2.3.3 Determination of Paste Yield

Accurately 10 ml of each batch of standard decoction was drawn and placed in an evaporating dish with constant weight (weigh the evaporating dish beforehand), evaporated to dryness in a water bath, dried at 105° for 3 h, and taken out; then it was cooled in a desiccator for 30 min, the weight was weighed, the paste rate was calculated, and the formula for calculating the paste rate was

Paste rate(%)=(w∗V)/(v∗M)∗100%,

where M is the number of medicinal materials (g), V is the volume of the standard decoction of Chinese herbal decoction pieces (ml), v is the sampling volume (ml), and w is the amount of dry paste obtained by sampling (g).

#### 2.3.4 Determination of pH Value

An appropriate amount of standard decoction was taken and the pH value was measured with a pH meter (Model PHS-3E, Shanghai, China).

#### 2.3.5 Study on the Characteristic Map of Standard Decoction

##### 2.3.5.1 Chromatographic Conditions

Chromatographic column: Agilent-ZORBAX SB-C18 column (250 mm × 4.6 mm, 5.0 μm); with water (A)-acetonitrile (B) as mobile phase; gradient elution; elution program: 0–10 min, 2%–10%B; 10–20 min, 10%–20%B; 20–40 min, 20%–25%B; 40–50 min, 25%–35%B; 50–60 min, 35%–50%B; 60–70 min, 50%–100%B. Flow rate: 0.5 ml/min; detection wavelength: 225 nm; column temperature: 25°C.

##### 2.3.5.2 Preparation of the Test Solution

Precisely 1 ml of *A. paniculata* standard decoction (CXL-01∼CXL-12) was pipetted into a 2-ml centrifuge tube and centrifuged at 12,000 rpm for 5 min. Then, the supernatant was taken and filtered with a 0.45-µm filter to get the test solution.

##### 2.3.5.3 Preparation of Reference Solution

Appropriate amounts of Ap and Neo reference substances, respectively, were taken and accurately weighed, and they were dissolved in methanol to prepare a mixed reference substance solution so that the concentration of each reference substance could be 100 μg/ml.

##### 2.3.5.4 Methodological Investigation

###### 2.3.5.4.1 Precision Test

The same *A. paniculata* standard decoction test solution (CXL-10) was taken, the sample injection was repeated 6 times according to the chromatographic conditions in Section 2.3.5.1, and 10 µl was injected. No. 4 peak was taken as the S peak, and the peak area and retention time of each common peak were measured.

###### 2.3.5.4.2 Repeatability Test

Six samples of *A. paniculata* standard decoction test solution (CXL-10) were prepared in parallel, and 10 µl was injected according to the chromatographic conditions in Section 2.3.5.1. No. 4 was taken as the S peak, and the peak area and retention time of each common peak were measured.

###### 2.3.5.4.3 Stability Test

*A. paniculata* standard decoction test solution (CXL-10) was taken, and 10 µl of the sample solution was injected at 0, 3, 6, 9, 12, and 24 h after the preparation of the test solution according to the chromatographic conditions in Section 2.3.5.1. As the S peak, the peak area and retention time of each common peak were measured.

##### 2.3.5.5 Establishment of Feature Map and Similarity Evaluation

According to the chromatographic conditions in Section 2.3.5.1, 10 μl of 12 batches of *A. paniculata* standard decoction solution for testing were accurately drawn, injected into a high-performance liquid chromatography, and the chromatographic peak information was recorded.

##### 2.3.5.6 Identification of Common Peaks

Precisely 10 μl each of 12 batches of *A. paniculata* standard decoction test solution was drawn and mixed with reference solution, injected into a high-performance liquid chromatography, and identified the main common peaks by comparing the retention time of the reference substance.

### 2.4 Statistical Analysis

SPSS 19.0 statistical software was used for all statistical analyses. The significant differences were analyzed using analysis of variance (ANOVA), and the *p* value less than 0.05 was considered to be statistically significant.

## 3 Results

### 3.1 Chromatography-MS Results

The information on the five reference parent ions and daughter ions is shown in Table 2 in the following text.

**TABLE 2:** Information of *A. paniculata* sample.

Serial number	Factory	Origin	Production batch
CXL-01	Jiangxi Zhangshu Tianqitang Traditional Chinese Medicine Co., Ltd.	Guangxi Province	1611004
CXL-02	Jiangxi Zhangshu Tianqitang Traditional Chinese Medicine Co., Ltd.	Guangxi Province	1702001
CXL-03	Jiangxi Zhangshu Tianqitang Traditional Chinese Medicine Co., Ltd.	Guangxi Province	1606002
CXL-04	Anguo Changda Prepared Chinese Medicinal Herbs Co., Ltd.	Fujian Province	1504001
CXL-05	Anguo Changda Prepared Chinese Medicinal Herbs Co., Ltd.	Fujian Province	1506001
CXL-06	Anguo Changda Prepared Chinese Medicinal Herbs Co., Ltd.	Fujian Province	1505001
CXL-07	Bozhou Huqiao Pharmaceutical Co., Ltd.	Anhui Province	1606110052
CXL-08	Jiangxi Jiangzhong Prepared Slices of Chinese Crude Drugs Co., Ltd.	Guangxi Province	160323
CXL-09	Anguo Changda Prepared Chinese Medicinal Herbs Co., Ltd.	Hubei Province	1701001
CXL-10	Anguo Changda Prepared Chinese Medicinal Herbs Co., Ltd.	Hubei Province	1706001
CXL-11	Anguo Changda Prepared Chinese Medicinal Herbs Co., Ltd.	Hubei Province	1703001
CXL-12	Harbin Runhe Chinese Herbal Pieces Processing Factory	Guangdong Province	16050401

### 3.2 Standard Curve Results

The standard curve equations and linear ranges of the six reference substances are detailed in Table 3 in the following text.

**TABLE 3:** Cream rate and pH value of standard decoction.

Serial number	Cream rate (%)	pH Value
CXL01	11.8	5.5
CXL02	17.5	5.9
CXL03	11.0	5.9
CXL04	13.0	5.5
CXL05	11.8	5.4
CXL06	11.5	5.5
CXL07	14.0	5.9
CXL08	18.3	6.0
CXL09	16.0	5.5
CXL10	15.2	5.5
CXL11	16.0	5.5
CXL12	16.0	5.8

### 3.3 The Effect of Ultraviolet Treatment on Chemical Composition of *A. paniculata* Seedlings

**FIGURE 1:**
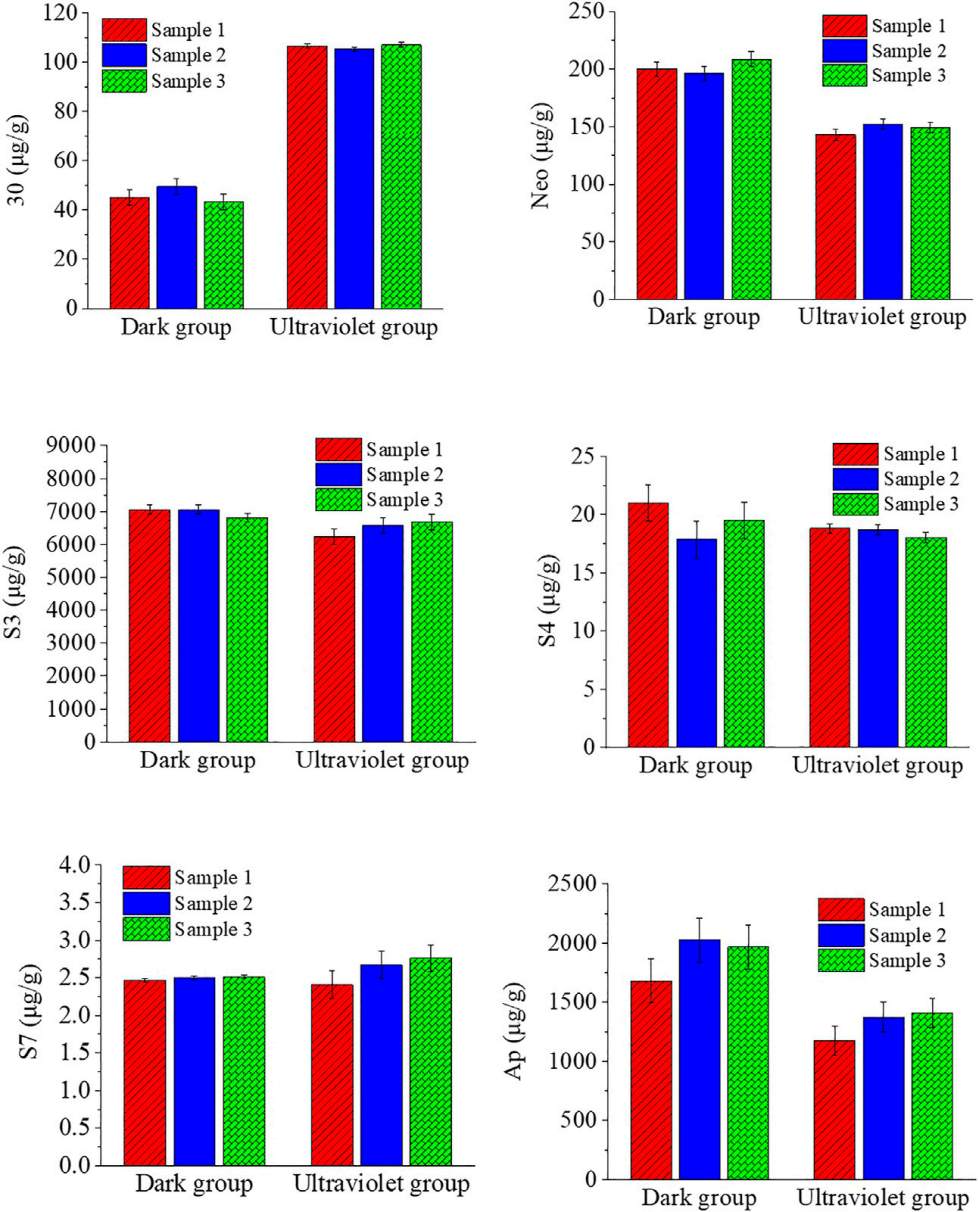
Effect of ultraviolet light treatment on the chemical composition of *A. paniculata* seedlings.

The effect of ultraviolet treatment on the chemical composition of *A. paniculata* seedlings is shown in Figure 1. It showed that UV treatment can indeed significantly increase the content of chemical substance 30 (roughly doubled), and the S7 content increased slightly, but the effect on other ingredients is negative or insignificant. The contents of Neo, S3, S4, and Ap in the UV group were reduced by 26.52%, 6.74%, 4.80%, and 30.19%, respectively, compared with the dark group. The total content of andrographolides is reduced by 11.3% compared with the dark group.

### 3.4 The Effect of Other Light Treatments on Chemical Composition of *A. paniculata* Seedlings

**FIGURE 2:**
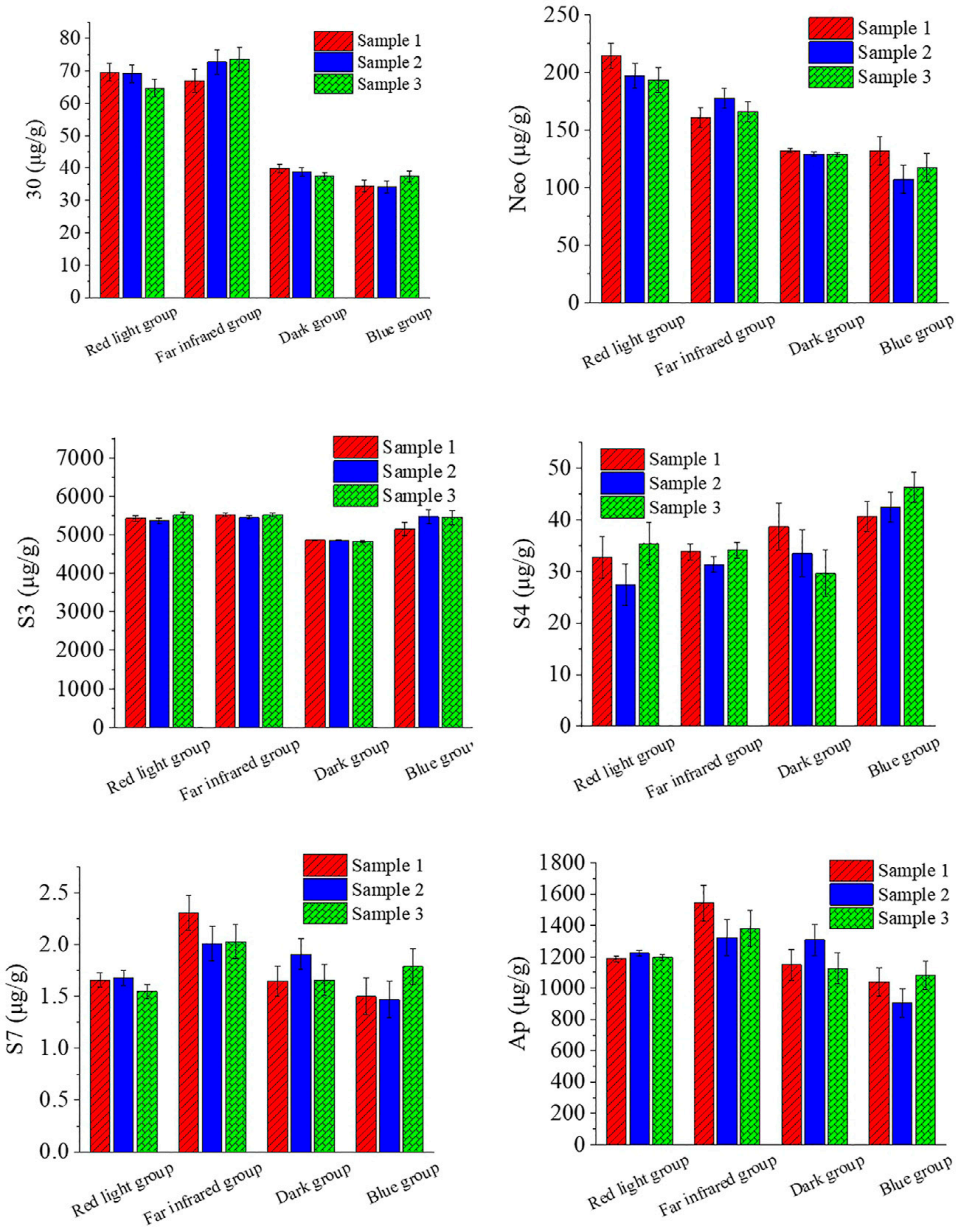
Effect of other light treatments on the chemical composition of *A. paniculata* seedlings.

The effect of other light treatments on the chemical composition of *A. paniculata* seedlings is shown in Figure 2.

For chemical composition 30, the content of the red light group and the far-infrared group was increased compared with the dark group; the increase ratio was 75.16% and 83.70%, while the content of the blue light group and the dark group was about the same, with little change.

For chemical composition Neo, the red light group content increased by 55.24% compared with the dark group, and the far-infrared group content increased by 29.34%. The difference between the blue light group and the dark group was small.

For chemical component S3, the content of the red light group, the far-infrared group, and the blue light group was higher than that of the dark group, and the increase ratios were 12.09%, 13.38%, and 10.44%, respectively.

For chemical component S4, the content of the blue light group was slightly more than that of the other three groups (the blue light group increased by about 27.19% compared to the dark group), while the red light group, the far-infrared group, and the dark group had little change.

For chemical composition S7, the red light group, the dark group, and the blue light group were similar in content, and the content of the far-infrared group was slightly more than that of the other three groups.

For chemical composition Ap, the red light group, the dark group, and the blue light group were similar in content, and the content of the far-infrared group was slightly more than that of the other three groups.

In summary, the effects of red and far-infrared light on 30, Neo, and S3 are relatively large, which can significantly increase the content of 30, Neo, and S3; blue light has a greater influence on S3, S4, and Ap, which can significantly increase the content of S3 and S4.

### 3.5 Determination of Paste Yield

The results of paste yield are shown in Table 4. The paste yield ranged from 11.0% to 18.3%, the average paste yield was 14.3%, and the relative standard deviation (RSD) value was 17.5%, with little variation between batches.

**TABLE 4:** Chromatograms’ similarity evaluation of standard decoction.

Sample	S1	S2	S3	S4	S5	S6	S7	S8	S9	S10	S11	S12	R
S1	1.000	1.000	0.999	0.984	0.980	0.982	0.996	0.995	0.985	0.987	0.989	0.991	0.996
S2	1.000	1.000	0.998	0.983	0.980	0.982	0.997	0.994	0.986	0.987	0.988	0.992	0.996
S3	0.999	0.998	1.000	0.978	0.973	0.975	0.995	0.994	0.979	0.982	0.984	0.986	0.993
S4	0.984	0.983	0.978	1.000	1.000	1.000	0.983	0.976	0.999	0.999	0.998	0.993	0.995
S5	0.980	0.980	0.973	1.000	1.000	1.000	0.980	0.972	0.999	0.998	0.997	0.993	0.993
S6	0.982	0.982	0.975	1.000	1.000	1.000	0.982	0.974	0.999	0.998	0.998	0.994	0.994
S7	0.996	0.997	0.995	0.983	0.980	0.982	1.000	0.994	0.987	0.989	0.990	0.994	0.996
S8	0.995	0.994	0.994	0.976	0.972	0.974	0.994	1.000	0.978	0.982	0.984	0.988	0.991
S9	0.985	0.986	0.979	0.999	0.999	0.999	0.987	0.978	1.000	0.999	0.999	0.997	0.996
S10	0.987	0.987	0.982	0.999	0.998	0.998	0.989	0.982	0.999	1.000	1.000	0.997	0.997
S11	0.989	0.988	0.984	0.998	0.997	0.998	0.990	0.984	0.999	1.000	1.000	0.997	0.997
S12	0.991	0.992	0.986	0.993	0.993	0.994	0.994	0.988	0.997	0.997	0.997	1.000	0.998
R	0.996	0.996	0.993	0.995	0.993	0.994	0.996	0.991	0.996	0.997	0.997	0.998	1.000

### 3.6 Determination of pH Value

The results of pH value are shown in Table 4. The pH value ranged from 5.4 to 6.0, the average pH value was 5.7, the RSD value was 3.8%, and the pH value difference between different batches was small.

### 3.7 Characteristic Map of Standard Decoction

#### 3.7.1 Precision Test

The results showed that the RSD of relative retention time and relative peak area of each common peak in the test solution of *Andrographis paniculatum* standard decoction were less than 5%, indicating that the precision of the instrument was good.

#### 3.7.2 Repeatability Test

The results showed that the RSD of the relative retention time and relative peak area of each common peak in the test solution of *A. paniculatum* standard decoction were less than 5%, indicating that the method had good repeatability.

#### 3.7.3 Stability Test

The results showed that the RSD of the relative retention time and the relative peak area of each common peak were all less than 5%, indicating that the *A. paniculata* standard decoction test solution had good stability within 24 h.

#### 3.7.4 Feature Map and Similarity Evaluation

**FIGURE 3:**
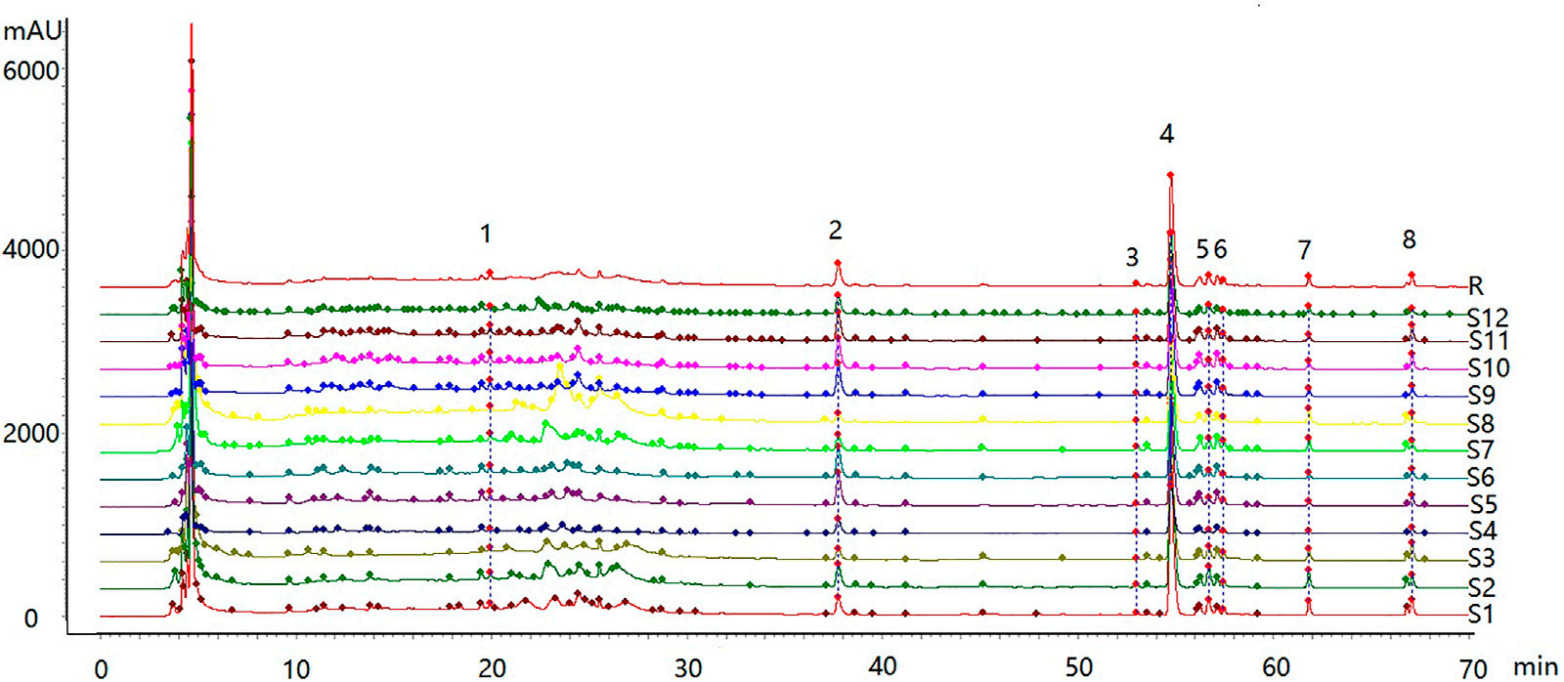
Characteristic map of 12 batches of *A. paniculata* standard decoction.

**FIGURE 4:**
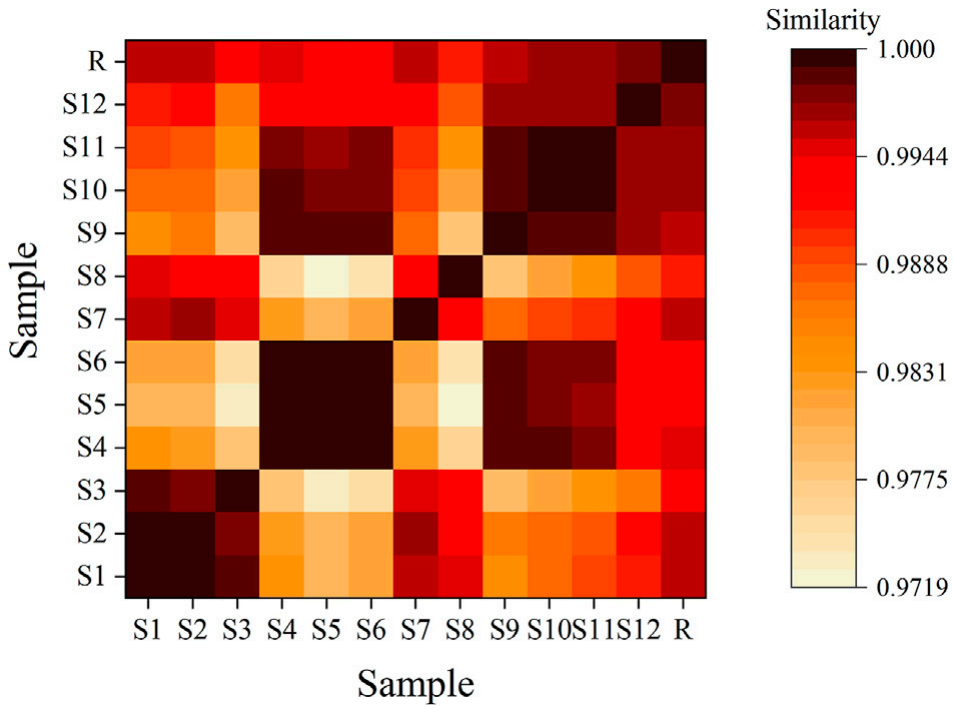
Chromatogram similarity heatmap for standard decoctions.

**FIGURE 5:**
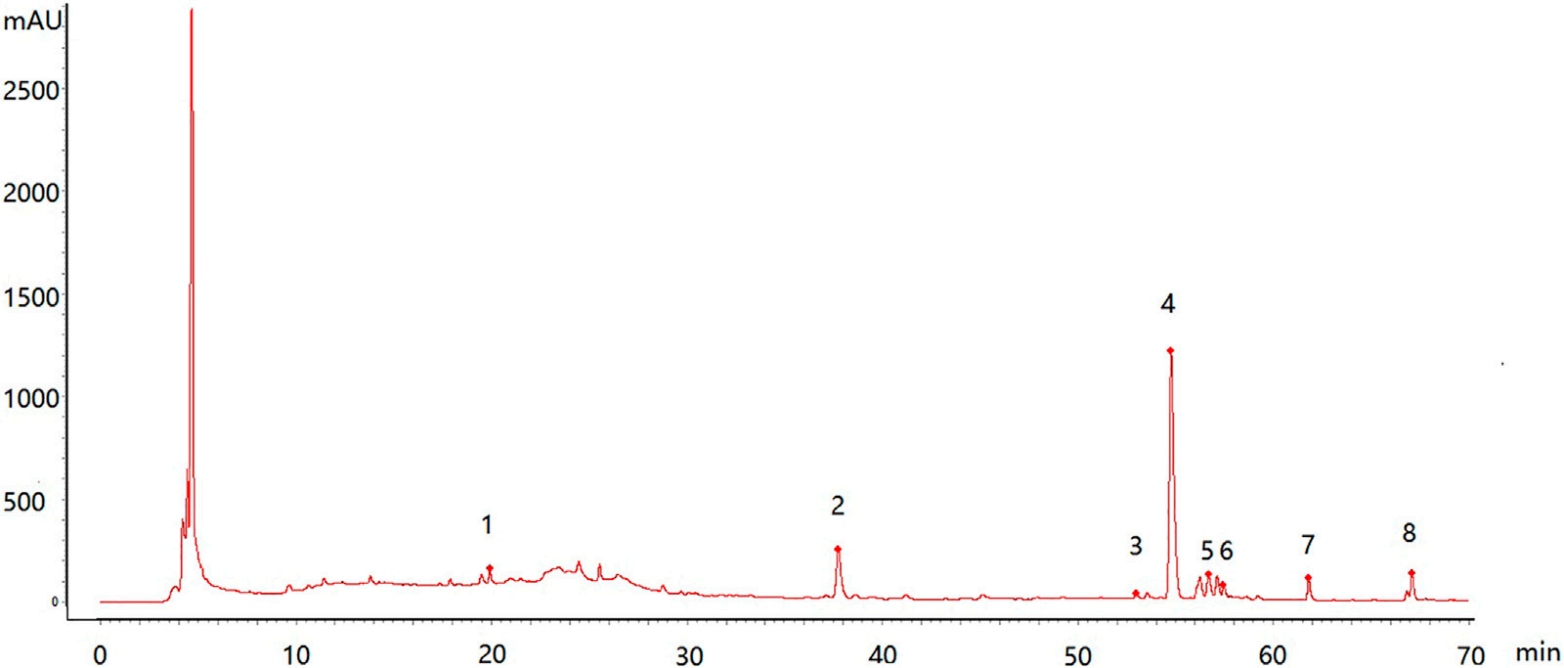
Comparison of characteristic spectra and confirmation of common peaks.

The feature map is shown in Figure 3, similarity results are shown in Figure 4, and the generated control feature map is shown in Figure 5. The results showed that the similarity of 12 batches of *A. paniculata* was greater than 0.95, the similarity was high, and eight peaks were identified. The peak area of each common peak is shown in Table 5. Taking peak No. 4 as the reference peak, the relative retention time and relative peak area of other common peaks are calculated (Table 6).

**TABLE 5:** Common peak area of each.

Serial number	Retention time	S1	S2	S3	S4	S5	S6	S7	S8	S9	S10	S11	S12	Control fingerprint
1	19.9	9.9	19.7	8.4	4.7	14.3	13.2	25.8	7.8	21.4	20.3	22.2	22.8	15.9
2	37.7	57.1	71.8	39.8	44.8	101.8	98.8	50.3	26.1	100.5	92.2	88.5	58.3	69.2
3	53.0	5.7	6.8	3.1	2.2	5.2	4.8	10.2	3.5	5.8	7.6	6.1	5.2	5.5
4	54.8	426.4	532.5	400.2	140.3	297.2	297.3	334.1	193.1	329.3	317.4	316.2	235.7	318.3
5	56.7	36.9	52.4	32.9	7.6	17.6	19.6	31.2	27.2	19.8	20.9	23.4	25.8	26.3
6	57.5	11.3	9.1	11.4	4.0	8.1	7.2	28.9	10.3	13.2	16.9	13.7	9.8	12.0
7	61.8	25.0	29.9	20.8	4.4	9.4	9.5	24.0	26.0	13.5	13.4	13.0	15.6	17.0
8	67.1	30.4	25.8	33.9	12.2	21.6	19.8	19.5	22.6	20.6	29.5	34.2	11.9	23.5

**TABLE 6:** Parameters of common peaks of standard decoction.

Serial number	Retention time	Relative retention time	Peak area	Relative peak area
1	19.910	0.364	15.875	0.050
2	37.741	0.689	69.170	0.217
3	52.989	0.968	5.515	0.017
4	54.760	1.000	318.307	1.000
5	56.676	1.035	26.270	0.083
6	57.460	1.049	11.994	0.038
7	61.793	1.128	17.040	0.054
8	67.078	1.225	23.491	0.074

#### 3.7.5 Identification of Common Peaks

Results: A total of two components were identified, of which the 4th peak was Ap and the 7th peak was Neo.

## 4 Discussion

Light quality is an important factor affecting the chemical composition of plants. Ebel et al. (1974) clarified that the synthetic pathways of flavonoids and lactones from photosynthetic carbohydrates to pentose phosphate have a common pathway, and only when erythrose 4-phosphate forms their own synthetic pathways. Therefore, Tang et al. (2020) concluded that UV-B pretreatment of plants in the seedling stage can affect the accumulation of phenolic acids and flavonoids, and has a high reference value for this experiment. In this study, the content of chemical component 30 in the ultraviolet light group is twice that of the dark group, and the content of chemical components Neo, S3, and Ap in the ultraviolet light group is slightly lower than that in the dark group. For chemical components S4 and S7, the content of the ultraviolet light group and the dark group does not change significantly.

By studying DWF4-GUS (a key brassinosteroid biosynthesis enzyme) marker plants grown in several monochromatic light conditions, Sakaguchi et al. (2019) revealed that at the same intensity of monochromatic light, monochromatic blue LED could induce DWF4 accumulation in primary root tips and root growth as much as white light, whereas monochromatic red LED could not. Consistent with this, a cryptochrome 1/2 double mutant showed retarded root growth under white light, whereas a phytochrome A/B double mutant did not. Taken together, their data strongly indicated that blue light signaling was important for DWF4 accumulation in root tips and root growth. Research of Wang and Xie (2006) on the lactone content in the ginkgo leaves treated with different wavelengths of projected light has similar results. These are consistent with the research results obtained in this experiment that blue light can significantly increase the content of S3 and S4.

During the investigation of different light qualities on the growth of *Epimedium pseudowushanense* by Yang et al. (2019), it was found that yellow light treatment had beneficial effects on shoot number, dry biomass (per plant) as well as net photosynthesis rate and maximal apparent quantum efficiency in *E. pseudowushanense* when compared with red or blue light treatment. More importantly, they found that *E. pseudowushanense* accumulated higher levels of bioactive flavonoids under yellow light treatment than under white, red, or blue light treatment. Correspondingly, this study shows that other light treatments can increase the content of total lactones in *A. paniculata* at different levels. Among them, red light, far-infrared light, and blue light groups increased by 11.09%, 15.06%, and 5.05%, respectively. In addition, under the conditions of red light and far-infrared light, chemical composition 30 is more sensitive than other ingredients, and its content changes significantly (roughly doubled). For chemical composition Neo, the content of the red light group and the far-infrared group is slightly higher than that of the dark group and the blue light group. Chemical component S3 is sensitive to red light, far-infrared light, and blue light, but the sensitivity is not high, and the increment is about 10%.

*A. paniculata* and its preparation quality detection methods are more reported, most of which are HPLC methods, and the measured components are especially andrographolide and anhydroandrographolide; these two components are the most, and there are also literatures to measure andrographolide and neoandrographolide; also, some literatures use andrographolide, anhydroandrographolide, and neoandrographolide as the index components for quality control, and some literatures establish an analytical method for the simultaneous determination of 4 or six lactones in *A. paniculata* extract. Andrographolide and anhydroandrographolide are the quality control index components of *A. paniculata*, but the 2015 edition of the Pharmacopoeia does not specify the method and index components for the content determination of *A. paniculata*. Therefore, it is difficult to guarantee the quality of the processed *A. paniculata*, and it is urgent to establish a simple, fast, stable, and reliable determination method to control its quality. For *A. paniculata* standard decoction, it is also necessary to determine the corresponding active ingredients in order to control the quality of the standard decoction. The common peaks of 12 batches of standard decoction were identified by comparison with reference substances, and it was found that peak No. 4 was andrographolide, and the content was relatively high compared with other components. It is recommended to select andrographolide as the index component of the standard decoction of *A. paniculata*. In this chapter, the research on the fingerprints of the standard decoction of *A. paniculata* is not deep enough. Only two components have been identified in the common peak, and other index components may be added. Decoction pieces and standard decoctions need to establish a method for determining the content of active ingredients. There are deficiencies, and further research is needed.

For the determination of the mobile phase of the fingerprint of the standard decoction of *A. paniculata*, it was found by comparison that the chromatogram generated by acetonitrile-water was better than the chromatogram generated by methanol-water, and the chromatographic peak resolution was higher, so the mobile phase was determined to be acetonitrile-water.

According to the analysis of the similarity evaluation system of traditional Chinese medicine chromatographic fingerprints (version 2012), it was determined that there were eight peaks in total, and two were identified. Compared with the control fingerprints, the fingerprints of each batch of *A. paniculata* standard decoction had a high similarity, which were all greater than 0.95. The pH range was 11.0%–18.3%, the average creaming rate was 14.3%, and the standard deviation was 2.5%; the pH range was 5.4–6.0, the average pH was 5.7, and the standard deviation was 0.2%. The results show that the established quality standard system of *A. paniculata* can provide a preliminary reference for the establishment of the quality standard of *A. paniculata*.

## 5 Conclusion

This experiment studied the effects of different wavelengths of light on the content of six active ingredients (Ap, Neo, 30, S3, S4, and S7) in the body of *A. paniculata* seedlings. The results showed that the UV treatment significantly increased the content of chemical component 30, which was twice that of the dark group. The changes of S4 and S7 were not significant, while the content of Neo, S3, and Ap was lower than that of the dark group. Red light and far-infrared light can significantly increase the content of 30, Neo, and S3, while the changes of S4, S7, and Ap are not significant. Blue light can significantly increase the content of S3 and S4, while the changes of 30, Neo, S7, and Ap are not significant. Using the ointment yield and pH value, the fingerprint analysis method of *A. paniculata* standard decoction was established, and we selected andrographolide as the index component of *A. paniculata* standard decoction. The establishment of the standard provides a reference.

This study, which shows the effects of different light qualities on the chemical constituents of medicinal plant *A. paniculata* seedlings, provides key information for the large-scale cultivation of *A. paniculata*, concurrently enhancing the accumulation of active ingredients and the suitable light quality of other medicinal plants.

## Data Availability

The original contributions presented in the study are included in the article/Supplementary Material; further inquiries can be directed to the corresponding authors.

## Abbreviations

Ap, andrographolide; Neo, neoandrographolide; 30, 14-deoxyandrographolide; S3, 14-deoxyandrographolide; S4, 14-deoxy-11-hydroxyandrographolide; S7, 14-deoxy-17-hydroxyandrographolide.
